# Miscellaneous Medical Intelligence

**Published:** 1851-11

**Authors:** 


### ARTICLE III.

### MISCELLANEOUS MEDICAL INTELLIGENCE.

We hear that there are over two hundred Medical Students in attendance at the University of Michigan at Ann Arbor.

The class in Rush Medical College, at the opening of the present Session is larger than ever before. The prospect is good for a very full class, as students are coming in rapidly. A large portion of those in attendance have already taken the Hospital ticket.

There is shortly to appear a new work on Surgical Anatomy and Operative Surgery, by M. M. Bernard and Huette of Paris, with 150 original steel plates.

During the year that preceded the taking of the census, the deaths in Vermont were one to every ninety persons ; in Rhode Island one to every sixty-six ; in South Carolina one to every forty-eight; and in Arkansas one to every fifty-four.

A new State Hospital for the Insane is about to be erected n Massachusetts. The Hospital provisions for the Insane in that State already exceed by nearly one half those of any other State in the Union.

The Army board of Medical examiners for Surgeons in the service of the United States, will be in session in the city of New York on the 20th instant.

Paul F. Eve, the former able editor of the Southern Medical and Surgical Journal, and late Prof. of Surgery in the Medical College of Georgia and the University of Louisville, has accepted the chair of Surgery in the University of Nashville, Tenn., and become associate editor of the Nashville Medical Journal.

A new Dental College is about to open in Syracuse, New York.

Dr. Farnham, convicted of conspiracy against the Michigan Central Railroad, and sentenced to the Penitentiary is not a physician. His title arises from the fact that he has been practicing dentistry. The man whom the papers style Dr. Fitch, implicated in the same crime was a farmer and never either laid any claim to, or received the title of Doctor, until after the trial commenced. The only physician indicted was found innocent and set at liberty.

We hear from various parts of the country that Dysentry has prevailed very extensively during the past summer and early part of autumn, as it did the year preceding. It has been quite a common type of disease for three successive years in Chicago, though not marked by any considerable mortality.

The following we extract from a letter from W. H. Martin, M. D., of Rushville, Ind.—

"We have had a great deal of Dysentery to contend with this season. Many cases were of a severe character, and strange as it may seem, we have lost but one case. We depended chiefly on Opium, and injections of Sol. Starch, Acet. Plumbi and Thebecaic tinct., assisted by hot straps, made by wringing flannel out of very hot water and then moistening the surface with Turpentine. Many of our cases were ushered in by very severe vomiting. We found nothing so prompt in arresting this condition of the stomach as small doses of the Proto Chlor. Mercury, say ½ grain, exhibited dry and washed down with a mouthful of Elm water, every 20 or 30 minutes, according to the severity of the vomiting and until it ceased. I do not now recollect a case that resisted this treatment; and I know of but one or two that were in the least ptyalized."

Dr. Charles W. Wright of Cincinnati, Ohio, says, boiling a little Slippery Elm bark, (*Cort. Ulmus Fulva,*) in oils and fats completely prevents their becoming rancid. It gives to fresh butter the flavor of the kernel of the Hickory nut.

Dr. James B. Coleman reports a case of re-production of the Mammay gland after excision, in the New Jersey Medical Reporter.

Dr. Hachenberg of Springfield, Ohio, reports a case in the Western Lancet, in which the application of touruiquets to all the extremities near the body speedily arrested a violent hem-optysis.

